# Coping Styles in Pregnancy, Their Demographic and Psychological Influences, and Their Association with Postpartum Depression: A Longitudinal Study of Women in China

**DOI:** 10.3390/ijerph17103654

**Published:** 2020-05-22

**Authors:** Min Yu, Wenjie Gong, Beck Taylor, Yiyuan Cai, Dong (Roman) Xu

**Affiliations:** 1 Xiangya School of Public Health, Central South University, Changsha 410083, China; yumin0930@csu.edu.cn; 2 Institute of Applied Health Research, University of Birmingham, Edgbaston, Birmingham B15 2TT, UK; R.Taylor.3@bham.ac.uk; 3 Department of Preventive Medicine and Maternity and Child Care, School of Public Health, Guizhou Medical University, Guiyang 550025, China; caiyy28@mail2.sysu.edu.cn; 4 Department of Medical Statistics, School of Public Health, Sun Yat-Sen University, Guangzhou 510275, China; 5 Sun Yat-sen Global Health Institute (SGHI), School of Public Health, Sun Yat-sen University, Guangzhou 510275, China; roman.xu@gmail.com

**Keywords:** coping style, longitudinal study, postpartum depression, pregnancy

## Abstract

We aimed to investigate the coping styles of Chinese pregnant women, identify factors associated with coping and further explore the effect of coping during pregnancy on postpartum depression. A longitudinal study was performed from early pregnancy to six-week postpartum. A total of 1126 women were recruited by convenience sampling and participants who completed eight questionnaires at four time points were included (three self-developed questionnaires, Coping Style Questionnaire, Generalized Anxiety Disorder-7, Brief Resilience Scale, Rosenberg Self-esteem Scale, Edinburgh Postnatal Depression Scale) (n = 615). Linear regression analyses were used to identify the possible factors for coping and their association with postpartum depression. The mean scores of positive coping and negative coping were 2.03 and 1.21, respectively. Women with a higher educational level scored higher on both positive and negative coping in pregnancy. Resilience was associated with both positive and negative coping, while self-esteem only related to positive coping (*p* < 0.05). Postpartum depression was associated with both positive and negative coping (*p* < 0.05). The women in our study reported using positive coping styles more than negative coping antenatally. Positive and negative coping behaviors could be used simultaneously. Increasing self-esteem and resilience antenatally might promote more positive coping and further reduce the occurrence of postpartum depression.

## 1. Introduction

Coping is defined as consciously and constantly changing behavioral and cognitive efforts to deal with the specific situations that are considered stressful [1]. Pregnancy and childbirth are often stressful life events for women [2,3], and women can experience a range of physical and psychological challenges. Pregnancy and birth-related stressors, for example back pain, gestational diabetes, renegotiating relationships, and fear of childbirth, could further lead to emotional problems [4]. In pregnancy, adopting an adaptive or appropriate coping style could minimize or even prevent the adversities brought by stressors [5]. Women taking an active coping style and resolving the problem would receive less adverse effects of stress, while those avoiding and adopting unhealthy behaviors like smoking would be more vulnerable [5]. Coping styles can be broadly classified into ‘positive’ and ‘negative’. Positive coping refers to the ability to handle problems, adapt quickly to stressors and moderate stress responses. Negative coping strategies, such as avoidance, social withdrawal, self-pity and self-accusation, could aggravate anxiety [6,7]. Guardino and Schetter undertook a review which concluded that avoidance coping strategies were associated with postpartum depression, preterm birth and poor infant outcomes, noting that cultural context might modify the impact of coping styles [5].

The choice of coping styles during pregnancy varies across different cultural contexts. Pregnant women in Japan benefited more from social-support coping than American women [8], and women with postpartum depression in rural Ethiopia were more likely to use emotion-focused and religious coping due to the popularity of religion [9]. Most studies on coping have been performed in western countries dominated by individualistic cultures (e.g., United States) and rarely in collectivist cultures (e.g., China) [10]. Coping-focused studies in Chinese women have concentrated on specific populations like first-time mothers [11,12] or women experiencing earthquake [13]. Studies of coping in the general pregnant population in Chinese women are still needed. Recent evidence has indicated that people can benefit greatly from coping interventions [14,15]. The identification of characteristics associated with Chinese women’s coping styles during pregnancy can inform the development, implementation and testing of targeted interventions to improve health and wellbeing for women and their babies.

We used a longitudinal study design to investigate women’s coping styles in pregnancy and to identify possible demographic and psychological factors associated with coping style. We further explored the association of coping styles with postpartum depression.

## 2. Materials and Methods

### 2.1. Study Design

This was a nested longitudinal study using data from a larger cohort study in Hunan Province, central south of China, the primary aim of which was to develop a dynamic predictive model of perinatal depression by measuring depression, social environment, psychological and biological factors from the first trimester of pregnancy to 6 weeks postpartum (not published).

### 2.2. Setting

This study took place in two urban maternal and child health hospitals in Hunan province, China: Hunan Maternal and Child Health Hospital (located in Changsha, the capital city of Hunan province) and Ziyang Maternal and Child Health Hospital (in a less economic developed area of Hunan province). Hunan Province is a medium-developed region in China with a per capita growth domestic product (GDP) of 49,558 Yuan (about 5486 GBP) in 2017 [16].

### 2.3. Participants and Recruitment

Pregnant women over the age of 18 under 13 weeks’ gestation (gestational weeks was calculated based on the last menstruation) were recruited to the main cohort study. The recruitment time was from September 2016 to February 2017. All women attending the hospital outpatient clinic for their antenatal appointment were approached by researchers individually and given detailed information concerning the study design and aim. Only women who gave their written informed consent and were willing to accept follow-up were included. There were no extra exclusion criteria. A total of 1126 women were recruited. All subjects gave their informed consent for inclusion before they participated in the study. The study was conducted in accordance with the Declaration of Helsinki, and the protocol was approved by the institutional review board of the institute of clinical pharmacology of Central South University (No. ChiCTR-ROC-16009255).

### 2.4. Data Collection

The study comprised of four time points for data collection (three time points prenatally and one point postpartum): gestational week 12 or earlier (range: week 4~12, T1); week 21~24 (T2); week 31~32 (T3); and 6 weeks postpartum (T4). Data were collected in two ways: questionnaires were transformed into online version using the smartphone application ’Wenjuanxing’, a questionnaire-survey platform [17]. At recruitment, we sent a questionnaire link to women using the WeChat App, a dominant mobile communication service in China [18], and women completed the survey on their phone when waiting for their antenatal check-up. Women unable to use their phone (e.g., network issues, insufficient battery) completed paper questionnaires. During the follow-up period, study administrators sent a link using the WeChat App to participants one week before the scheduled perinatal check-up requesting them to complete surveys online. Nurses at the two participating hospitals provided paper questionnaires to women who did not complete the online survey prior to their appointments, which women completed in the waiting room. Overall, 88.7% of the women in our study completed the surveys online.

**Figure 1:**
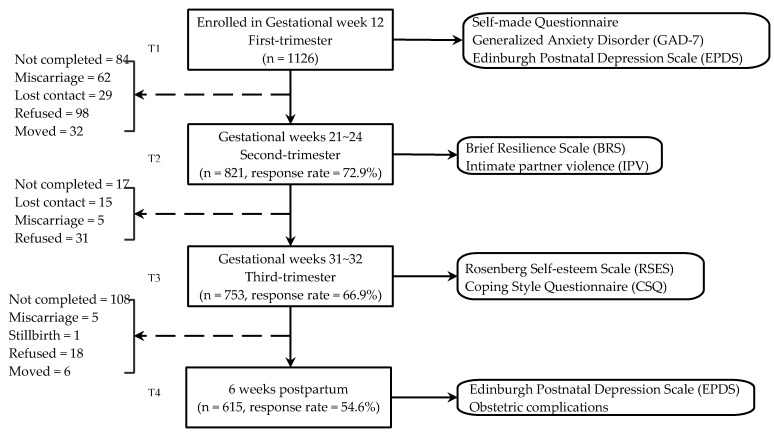
Flow of participants through the study.

Information about recruitment and participants flow is depicted in Figure 1. Coping and its potential influencing factors were detected in T1, T2, and T3. Depression at T4 was an outcome variable in the study of relationship between coping and postpartum depression (see Figure 1). At the end of the data collection period, 615 women who had completed questionnaires at T1, T2, T3, T4 constituted the participants in this study (response rate = 54.6%).

### 2.5. Measures

#### 2.5.1. Coping Styles

The Coping Style Questionnaire (CSQ) revised by Xie [7] was used in this study to measure the coping style of women. This scale was developed to suit the characteristics of Chinese people with satisfactory reliability and validity [7]. CSQ has two dimensions (positive coping and negative coping) and 20 items. The first 12 items correspond to positive coping and the others correspond to negative coping. By assigning scores of 0, 1, 2, and 3 to the four options of ‘never‘, ‘sometimes’, ‘often’, and ‘almost always’, respectively, the mean of each item could be calculated for each dimension and a higher score suggests a more frequent use of this coping style. The Cronbach’s alpha scores of positive and negative coping in this study were 0.86 and 0.77, respectively, suggesting the relatively high internal consistency of our data.

#### 2.5.2. Demographic Information

A questionnaire was developed by the researchers with simple items to investigate general demographic information including age, women’s income level (Yuans per month), marital satisfaction, current substance use (smoking and alcohol drinking). The variable, past history of any mood disorders, was assessed using two items (“have you ever been diagnosed with depression?” and “have you ever been diagnosed with any other mood disorders except depression like bipolar disorders?”) and women who endorsed any of the two items were considered to have past history of any mood disorders (see Table 1).

#### 2.5.3. Intimate Partner Violence

Intimate partner violence (IPV) was measured by four items (my partner punched or kicked me/my partner insulted or yelled at me/my partner used force to have sex with me or insisted on sex without a condom/my partner used to ignore me for a long time), which were adopted from the victimization subscale of the Short Form of the Revised Conflict Tactics Scale (CTS2S) and it shows good reliability and validity [19]. Women who endorsed any of the four items were considered as having IPV experience.

#### 2.5.4. Anxiety

Anxiety was measured by the Generalized Anxiety Disorder (GAD-7). GAD-7 compiled by Sptizer in 2006 [20] has good reliability and validity [20]. It had been widely used for screening anxiety disorders or evaluating the severity of anxiety symptoms in China with good reliability and validity [21,22]. Cronbach’s alpha in this study was 0.85.

#### 2.5.5. Resilience

Resilience, the ability to bounce back or recover from stress, was measured with the six-item Brief Resilience Scale (BRS), developed by Smith in 2008 [23]. We used the Chinese version of the BRS with good reliability and validity in our study [24], and Cronbach’s alpha in this study was 0.75.

#### 2.5.6. Self-Esteem

Self-esteem was assessed using Rosenberg Self-esteem Scale (RSES) [25] by asking the respondents to reflect on their current feelings about themselves. The Chinese version of RSES is considered a reliable and valid quantitative tool for self-esteem assessment and was used in our research [26,27]. Cronbach’s alpha of RSES was 0.79 in our study.

#### 2.5.7. Obstetric Complications

The obstetric complications were measured using one item (have you experienced any obstetric complications like postpartum hemorrhage, fetal distress or premature rupture of membranes during childbirth?)

#### 2.5.8. Antenatal and Postpartum Depression

Antenatal and postpartum depression was measured with the Edinburgh Postnatal Depression Scale (EPDS) developed by Cox [28]. The EPDS was originally developed for screening postnatal depression, but it has been validated for use in the antenatal period [29]. The scale is a 10-item self-report questionnaire asking participants to consider various depressive symptoms during the last 7 days. In our study, the verified Mainland Chinese version with satisfying psychometric validity of the EPDS was used and a score of 10 was taken as the cut-off point for ‘possible depression’ [30,31,32]. Cronbach’s alpha in this study were 0.85~0.88.

### 2.6. Statistical Analysis

All statistical analyses were performed using SPSS version 25.0. For sample description, mean and SD were calculated for continuous variables while the absolute number and composition ratio were used for categorical variables. Simple and multiple regressions were conducted to explore the potential factors of each of the two dimensions of coping styles. Multicollinearity was detected using tolerance and the variance inflation factor (VIF). Tolerance ≥0.1 or VIF ≥10 were considered as having multicollinearity [33]. When exploring the effects of coping on postpartum depression, a multiple linear regression was performed after adjusting for possible confounding factors which were associated with coping and also depression in this study. The statistical level of multivariate analyses was *p* < 0.05 (two-tailed).

## 3. Results

### 3.1. Characteristics of Participants

Participants with complete data constituted our study sample (n = 615). The mean age of participants was 30.74 (SD: 4.34; range: 20–46 years), and gestational age was from 4 to 12 weeks. There were no significant differences for most baseline characteristics between our study sample and the participants lost-to-follow-up for any of the surveys, with the exception of satisfaction in marriage, past history of mood disorders, and nulliparity (first pregnancy), which were more common in the study sample (Table 1).

### 3.2. Coping Styles

The mean scores of positive coping and negative coping were 2.03 (SD: 0.50), and 1.21 (SD: 0.51) respectively, indicating that the women in our study preferred using positive coping styles compared to negative coping during pregnancy. Among specific coping behaviors in positive coping, the most frequently used behaviors were “Focus on the good side of things” (50.2%) and “Talk with others about my trouble” (44.1%) (See Table 2).

### 3.3. Simple and Multiple Linear Regression Including Factors Related to Coping Styles

Multicollinearity was detected within two variables, antenatal depression and anxiety, which had tolerance = 0.86~0.89 or VIF = 10.47~11.08 in both multiple linear regression models. We thus excluded the variable of antenatal depression in analysis.

From Table 3, education status, resilience, and self-esteem showed statistically significant associations in both univariate and multivariate analysis. The positive regression coefficients of education status, resilience and self-esteem indicated that women having higher educational background, higher self-esteem or better mental resilience had higher scores of positive coping.

From Table 4, the education status of women was the only demographic variable significantly associated with negative coping, while having a bachelor’s degree was marginally associated with negative coping (*p* = 0.052). The positive regression coefficients of education status suggested that women with a higher educational level had higher scores of negative coping. Women who had better resilience had lower scores of negative coping.

### 3.4. Correlation between Coping Style and Postpartum Depression

The incidence of postpartum depression was 26.34% (n = 162). The characteristics of women with or without postpartum depression are listed in Table 5. A multiple linear regression was undertaken to explore the relationship between coping styles and six-week postpartum depression (see Table 6), and in this model, coping style still showed statistical significance after controlling for potential confounders. Those variables associated with coping and depression in univariate analyses were included in the model as potential confounders. Women with a higher level of positive coping in the third trimester were less likely to have postpartum depression (β = −0.560, *p* = 0.023), while women who had a higher level of negative coping were more likely to have postpartum depression (β = 0.491, *p* = 0.032).

## 4. Discussion

In this study, we found that women reported using positive coping styles more than negative coping styles during pregnancy. Education status and resilience were associated with both positive and negative coping while self-esteem only related to positive coping. Women with postpartum depression scored lower in positive coping and higher in negative coping.

Women in our study were inclined to use positive coping styles, and among specific coping behaviors within positive coping, the most frequently used behaviors were “Focus on the good side of things” and “Talk with others about my trouble”. This reflects the collectivistic cultures in China, in which people value group membership and tend to seek support or comfort from in-group members like friends, family members when facing problems or crises [34]. This aligns with observations in Japanese women, and may differ compared to women in more individualistic cultures in western countries which focus on emotional independence and being less attached to others emotionally [8,35]. The behaviors reported by women in our study also reflect an emotion-focused coping style which includes searching for comfort or understanding from others and altering one’s evaluation of the existing situation [5]. Understanding the coping characteristics identified in this study may assist care providers to develop culturally appropriate interventions to support women in non-western contexts.

Women with a higher educational level scored higher in both positive and negative coping. A study in Ethiopia had similar findings: compared with women without formal education, those with formal education had higher scores both in problem- and emotion-focused coping [9]. The association of better educational background with higher levels of positive coping might be attributed to women’s access to resources. Women with lower educational levels were more likely to perceive greater stress [36], which would affect the way they respond to stressors, and weaken their abilities to deal with challenges. Interestingly, we did not hypothesize that negative coping is associated with higher education status. One possibility is that positive and negative coping were not mutually exclusive, suggesting that they could be used simultaneously. This raises the question of whether the integration of different coping behaviors (rather than utilizing one particular coping behavior) might facilitate women’s capacity to handle pregnancy-related stressors: that individuals adapting better to stressful events might be exactly those who could combine different coping strategies effectively.

Women with better resilience were more likely to have a higher level of positive coping and lower level of negative coping in our study. Resilience refers to the ability to bounce back and recover from stress, or to continue forward when facing adversities [23]. Although few studies have explored the relationship between resilience and coping during pregnancy, existing research suggests that optimism and self-efficacy, as resilience resources [37], are negatively related with active coping [38] and the more resilience resources women have, the better they cope with stressful pregnancy-related events [37]. That is similar with our findings. Moreover, higher self-esteem was associated with increased use of positive coping, which concurs with a qualitative interview study undertaken with HIV-positive pregnant women in South Africa, which concluded that the increased use of active coping was associated with higher self-esteem and social supports [39].

Our findings indicate that women with a lower level of positive coping and higher level of negative coping in the antenatal period are likely to be more vulnerable to experiencing postpartum depression. According to coping theory, coping with stressors positively means mobilizing internal forces or resorting to external resources to minimize the adverse effects caused by stressors [5]. Conversely, women adopting negative coping might be vulnerable to prenatal stress exposures, which could promote the generation of negative emotion [5]. This result aligns with study of Ren et al., though the former targeted pregnant women who had experienced the earthquake in Ya’an city in 2013 [13]. Identifying coping styles and risk factors in the antenatal period, and implementing interventions for women at risk, may reduce the occurrence of postpartum depression.

There are a number of limitations in this research. First, 45.38% of participants were lost to follow-up during the study, potentially impacting on both internal and external validity. However, baseline characteristics between the study sample and the participants lost-to-follow-up were largely similar, suggesting that our sample was representative of the population of women attending the hospital. Second, most participants (76%) in our research were from urban contexts, and it is not clear how they apply in more rural communities. Third, based on ethical considerations, for women with EPDS >12 (major depression), we informed them of their risk for depression and suggested them to seek professional help. Our action, however, may affect the natural process of depression, although the effect should be minor, as only one woman (0.4%) accepted our advice. The integration of different coping strategies could be further explored in future research.

## 5. Conclusions

Chinese women in our study tended to adopt positive coping styles, in which focusing on the good side of things and talking with others were the most frequent behaviors they took, which reflects the characteristic of collectivistic cultures in China. This may imply the potential of developing culturally appropriate coping interventions in non-western contexts and enhancing women’s capacity to use more positive coping. Our findings also indicate that positive and negative coping strategies may be used simultaneously, and the integration of different coping strategies could be further explored in future research. Women with low self-esteem and resilience appear to be particularly at risk of coping difficulties. Increasing self-esteem and resilience antenatally might promote more positive coping and further potentially reduce their risk of depression in the postpartum period.

## Figures and Tables

**Table 1:** Comparison of baseline characteristics between the participants with complete data and those lost to follow-up.

Characteristics	Participants in Study (n = 615)		Participants Lost to Follow-Up (n = 511)		
	n	%	n	%	*p* Value ^1^
Advanced Age					
No	495	80.5	411	80.4	0.981
Yes	120	19.5	100	19.6	
Employed					
No	157	25.5	122	23.9	0.522
Yes	458	74.5	389	76.1	
Education status					
Middle school or lower	57	9.3	70	13.7	0.107
High school	141	22.9	117	22.9	
Bachelor	366	59.5	281	55.0	
Postgraduate or higher	51	8.3	43	8.4	
Income level					
<2000	200	32.5	168	32.9	0.099
2000~	322	52.4	224	43.8	
5000~	80	13	101	19.8	
≥10,000	13	2.1	18	3.5	
Marital satisfaction					
Very satisfied	518	84.2	405	79.3	0.031
Not very satisfied	97	15.8	106	20.7	
Living with parents-in-law					
No	411	66.8	332	65.0	0.512
Yes	204	33.2	179	35.0	
Past history of mood disorders					
No	598	97.2	506	99.0	0.031
Yes	17	2.8	5	1.0	
First pregnancy					
No	433	70.4	403	78.9	0.001
Yes	182	29.6	108	21.1	
Previous miscarriage					
No	305	49.6	269	52.6	0.308
Yes	310	50.4	242	47.4	
Parity					
0	362	58.9	277	54.2	0.117
≥1	253	41.1	234	45.8	
Smoking					
No	594	96.6	489	95.7	0.438
Yes	21	3.4	22	4.3	
Drinking					
No	568	92.4	472	92.4	0.995
Yes	47	7.6	39	7.6	
Employed (partner)					
No	17	2.8	10	2.0	0.378
Yes	598	97.2	501	98.0	
Education status (partner)					
Middle school or lower	73	11.9	71	13.9	0.814
High school	146	23.7	113	22.1	
Bachelor	343	55.8	281	55.0	
Postgraduate or higher	53	8.6	46	9.0	
GAD-7 (Mean, SD)	2.899 (2.862)		3.147 (2.941)		0.102
Depression (Mean, SD)	8.424 (3.992)		8.787 (3.965)		0.127

^1^*p*-value was calculated using c^2^ tests, *t*-tests and Mann–Whitney *u* tests. GAD-7: Generalized Anxiety Disorder.

**Table 2:** Frequency of use of positive and negative coping styles (n = 615).

Coping Styles	Mean (SD)	Never	Sometimes	Often	Almost Always
Positive Coping					
Take my mind off things through study, work or other activities	1.98 (0.79)	15 (2.4)	152 (24.7)	280 (45.5)	168 (27.3)
Talk with others about my trouble	2.21 (0.81)	7 (1.1)	129 (21.0)	208 (33.8)	271 (44.1)
Focus on good aspects of things	2.36 (0.73)	4 (0.7)	80 (13.0)	222 (36.1)	309 (50.2)
Change my mind and rediscover important things in life	2.13 (0.74)	4 (0.7)	119 (19.3)	282 (45.9)	210 (34.1)
Do not take problems too seriously	2.18 (0.83)	16 (2.6)	118 (19.2)	220 (35.8)	261 (42.4)
Stand my ground and fight for what I want	1.90 (0.80)	27 (4.4)	146 (23.7)	302 (49.1)	140 (22.8)
Come up with strategies to cope	2.06 (0.74)	8 (1.3)	129 (21.0)	298 (48.5)	180 (29.3)
Get help and advice from other people	2.08 (0.82)	25 (4.1)	108 (17.6)	274 (44.6)	208 (33.8)
Change some of my original practices or some of my own problems	1.90 (0.72)	14 (2.3)	149 (24.2)	337 (54.8)	115 (18.7)
Learn from others to deal with the similar situation	1.92 (0.76)	20 (3.3)	147 (23.9)	312 (50.7)	136 (22.1)
Seeking hobbies like taking part in cultural or sports activities	1.80 (0.85)	39 (6.3)	181 (29.4)	261 (42.4)	134 (21.8)
Try to control my disappointment, regret, sadness and anger	2.01 (0.80)	21 (3.4)	129 (21.0)	286 (46.5)	179 (29.1)
Negative coping					
Take a rest or vacation, put the problem aside temporarily	1.92 (0.84)	36 (5.9)	133 (21.6)	288 (46.8)	158 (25.7)
Use alcohol, cigarette or other drugs to help me get through it	0.20 (0.58)	541 (88.0)	35 (5.7)	31 (5.0)	8 (1.3)
Time will change the situation and the only thing I could do is waiting	1.15 (0.84)	131 (21.3)	308 (50.1)	130 (21.1)	46 (7.5)
Try to forget everything	1.25 (0.90)	131 (21.3)	261 (42.4)	162 (26.3)	61 (9.9)
Rely on others to solve problems	0.87 (0.77)	218 (35.4)	275 (44.7)	109 (17.7)	13 (2.1)
Accept the reality	1.27 (0.83)	105 (17.1)	287 (46.7)	175 (28.5)	48 (7.8)
Imagine the occurrence of miracle	0.98 (0.83)	191 (31.1)	273 (44.4)	125 (20.3)	26 (4.2)
Comfort myself	1.89 (0.83)	22 (3.6)	182 (29.6)	250 (40.7)	161 (26.2)

**Table 3:** Influencing factors of positive coping.

Characteristics	Mean (SD)	Crude β (95% CI)	Adjusted β (95% CI)
Advanced Age			
No	2.029 (0.503)	—	—
Yes	2.040 (0.510)	0.011 (−0.090, 0.112)	−0.003 (−0.108, 0.101)
Employed			
No	1.956 (0.529)	—	—
Yes	2.058 (0.494)	0.102 (0.011, 0.193)	−0.045(−0.163, 0.074)
Education status			
Middle school or lower	1.692 (0.571)	—	—
High school	1.974 (0.509)	0.283 (0.132, 0.433)	0.210 (0.056, 0.364)
Bachelor	2.077 (0.479)	0.385 (0.249, 0.522)	0.242 (0.083, 0.400)
Postgraduate or higher	2.245 (0.392)	0.553 (0.368, 0.738)	0.353 (0.134, 0.576)
Income level			
<2000	1.939 (0.519)	—	—
2000~	2.062 (0.494)	0.123 (0.035, 0.212)	0.063 (−0.051, 0.177)
5000~	2.101 (0.472)	0.162 (0.032, 0.292)	0.032 (−0.119, 0.183)
≥10,000	2.276 (0.526)	0.337 (0.056, 0.618)	0.172 (−0.100, 0.444)
Marital satisfaction			
Very satisfied	2.063 (0.501)	—	—
Not very satisfied	1.863 (0.492)	−0.200 (−0.309, −0.091)	−0.037 (−0.146, 0.072)
Living with parents-in-law			
No	2.049 (0.494)	—	—
Yes	1.997 (0.525)	−0.051 (−0.136, 0.034)	0.041 (−0.042, 0.123)
Past history of mood disorders			
No	2.029 (0.503)	—	—
Yes	2.123 (0.542)	0.094 (−0.149, 0.338)	0.053 (−0.175, 0.281)
First pregnancy			
No	1.989 (0.512)	—	—
Yes	2.132 (0.472)	0.143 (0.056, 0.230)	0.076 (−0.040, 0.193)
Previous miscarriage			
No	2.074 (0.494)	—	—
Yes	2.046 (0.477)	−0.083 (−0.163, −0.004)	0.006 (−0.090, 0.103)
Parity			
0	2.089 (0.484)	—	—
≥1	1.949 (0.522)	−0.140 (−0.221, −0.060)	−0.039 (−0.134, 0.057)
Smoking			
No	2.033 (0.504)	—	—
Yes	1.979 (0.511)	−0.054 (−0.274, 0.166)	−0.019 (−0.242, 0.204)
Drinking			
No	2.038 (0.507)	—	—
Yes	1.955 (0.471)	−0.083 (−0.233, 0.067)	−0.043 (−0.195, 0.109)
Employed (partner)			
No	1.897 (0.500)	—	—
Yes	2.035 (0.504)	0.138 (−0.105, 0.382)	0.050 (−0.178, 0.278)
Education status (partner)			
Middle school or lower	1.853 (0.488)	—	—
High school	1.927 (0.538)	0.075 (−0.065, 0.214)	−0.028 (−0.168, 0.113)
Bachelor	2.087 (0.485)	0.235 (0.109, 0.360)	0.077 (−0.063, 0.218)
Postgraduate or higher	2.206 (0.438)	0.354 (0.179, 0.529)	0.111 (−0.092, 0.314)
Intimate partner violence			
No	2.050 (0.496)	—	—
Yes	1.866 (0.548)	−0.082 (−0.149, −0.015)	−0.082 (−0.209, 0.044)
GAD−7 (Mean, SD)	2.899 (2.862)	−0.034 (−0.048, −0.020)	−0.013 (−0.027, 0.001)
Resilience (Mean, SD)	3.498 (0.580)	0.293 (0.228, 0.358)	0.233 (0.163, 0.302)
Self-esteem (Mean, SD)	29.385 (3.383)	0.024 (0.007, 0.040)	0.017 (0.001, 0.032)

GAD-7: Generalized Anxiety Disorder.

**Table 4:** Influencing factors of negative coping.

Characteristics	Mean (SD)	Crude β (95% CI)	Adjusted β (95% CI)
Advanced Age			
No	2.029 (0.503)	—	—
Yes	2.040 (0.510)	0.001 (−0.102, 0.104)	−0.008 (−0.125, 0.108)
Employed			
No	1.956 (0.529)	—	—
Yes	2.058 (0.494)	0.014 (−0.079, 0.107)	−0.010 (−0.141, 0.122)
Education status			
Middle school or lower	1.033 (0.502)	—	—
High school	1.233 (0.542)	0.200 (0.042, 0.357)	0.203 (0.032, 0.374)
Bachelor	1.214 (0.490)	0.181 (0.038, 0.324)	0.174 (−0.002, 0.351)
Postgraduate or higher	1.294 (0.580)	0.261 (0.067, 0.454)	0.267 (0.021, 0.513)
Income level (per month)			
<2000	1.186 (0.521)	—	—
2000~	1.217 (0.508)	0.031 (−0.060, 0.122)	0.005 (−0.123, 0.132)
5000~	1.198 (0.489)	0.012 (−0.122, 0.145)	−0.038 (−0.206, 0.129)
≥10,000	1.375 (0.683)	0.189 (−0.100, 0.478)	0.235 (−0.068, 0.537)
Marital satisfaction			
Very satisfied	2.063 (0.501)	—	—
Not very satisfied	1.863 (0.492)	0.069 (−0.042, 0.181)	0.020 (−0.101, 0.141)
Living with parents-in-law			
No	1.214 (0.514)	—	—
Yes	1.197 (0.513)	−0.017 (−0.103, 0.070)	0.012 (−0.079, 0.104)
Past history of mood disorders			
No	2.029 (0.503)	—	—
Yes	2.123 (0.542)	−0.017 (−0.266, 0.231)	−0.033 (−0.286, 0.221)
First pregnancy			
No	1.989 (0.512)	—	—
Yes	2.132 (0.472)	−0.030 (−0.119, 0.059)	−0.015 (−0.144, 0.114)
Previous miscarriage			
No	2.074 (0.494)	—	—
Yes	2.046 (0.477)	0.040 (−0.041, −0.121)	0.044 (−0.063, 0.151)
Parity			
0	2.089 (0.484)	—	—
≥1	1.949 (0.522)	−0.005 (−0.088, 0.077)	−0.004 (−0.110, 0.103)
Smoking			
No	2.033 (0.504)	—	—
Yes	1.979 (0.511)	−0.020 (−0.244, 0.204)	−0.034 (−0.282, 0.214)
Drinking			
No	2.038 (0.507)	—	—
Yes	1.955 (0.471)	0.058 (−0.095, 0.211)	0.074 (−0.095, 0.243)
Employed (partner)			
No	1.897 (0.500)	—	—
Yes	2.035 (0.504)	−0.036 (−0.284, 0.213)	−0.030 (−0.284, 0.223)
Education status (partner)			
Middle school or lower	1.116 (0.514)	—	—
High school	1.171 (0.510)	0.055 (−0.089, 0.199)	−0.006 (−0.163, 0.150)
Bachelor	1.235 (0.500)	0.119 (−0.011, 0.249)	0.072 (−0.084, 0.229)
Postgraduate or higher	1.258 (0.596)	0.142 (−0.040, 0.323)	0.073 (−0.153, 0.299)
Intimate partner violence			
No	1.198 (0.508)	—	
Yes	1.298 (0.554)	0.112 (−0.026, 0.250)	0.040 (−0.100, 0.181)
GAD-7 (Mean, SD)	2.899 (2.862)	0.014 (0.000, 0.028)	0.008 (−0.008, 0.023)
Resilience (Mean, SD)	3.498 (0.580)	−0.108 (−0.178, −0.039)	−0.106 (−0.183, −0.028)
Self-esteem (Mean, SD)	29.385 (3.383)	0.000 (−0.017, 0.017)	0.001 (−0.017, 0.018)

GAD-7: Generalized Anxiety Disorder.

**Table 5:** Characteristics between women with or without postpartum depression.

Variables	Without Postpartum Depression (n = 453) n (%) Mean (SD)	With Postpartum Depression (n = 162) n (%) Mean (SD)	*p* Value ^1^
Advanced Age			
No	357 (72.1)	138 (27.9)	0.079
Yes	96 (80.0)	24 (20.0)	
Employed			
No	100 (63.7)	75 (36.3)	0.001
Yes	353 (77.1)	105 (22.9)	
Education status			
Middle school	38 (66.7)	19 (33.3)	0.443
High school	103 (73.0)	38 (27.0)	
Bachelor	271 (74.0)	95 (26.0)	
Postgraduate or higher	41 (80.4)	10 (19.6)	
Income level			
<2000	137 (68.5)	63 (31.5)	0.010
2000~5000	242 (75.2)	80 (24.8)	
5001~10,000	61 (76.3)	19 (23.7)	
>10,000	13 (100)	0 (0)	
Marital satisfaction			
Very satisfied	396 (76.4)	122 (23.6)	0.001
Not very satisfied	57 (58.8)	40 (41.2)	
Past history of mood disorders			
No	442 (73.9)	156 (26.1)	0.735
Yes	11 (64.7)	6 (35.3)	
First pregnancy			
No	319 (73.7)	114 (26.3)	0.991
Yes	134 (73.6)	48 (26.4)	
Previous miscarriage			
No	219 (71.8)	86 (28.2)	0.300
Yes	234 (75.5)	76 (24.5)	
Parity			
0	261 (72.1)	101 (27.9)	0.294
≥1	192 (75.9)	61 (24.1)	
Intimate partner violence			
No	421 (76.1)	132 (23.9)	0.001
Yes	32 (51.6)	30 (48.4)	
Obstetric complications			
No	392 (66.0)	202 (34.0)	0.698
Yes	13 (61.9)	8 (38.1)	
GAD-7	2.488 (2.595)	4.048 (3.244)	0.001
Positive coping	2.086 (0.470)	1.878 (0.564)	0.001
Negative coping	1.183 (0.504)	1.278 (0.535)	0.042

^1^*p*-value was calculated using c^2^ tests and *t*-tests. GAD-7: Generalized Anxiety Disorder.

**Table 6:** Multivariate analysis of the association between coping and postpartum depression.

Variables	*B*	*St*	*β*	*t*	*p*
Advanced age	−0.461	0.488	−0.039	−0.946	0.345
Employed	−0.802	0.558	−0.075	−1.437	0.151
Education status					
Middle school	—	—	—	—	—
High school	−0.267	0.692	−0.024	−0.385	0.700
Bachelor	−0.077	0.666	−0.008	−0.115	0.908
Postgraduate or higher	−0.041	0.915	−0.002	−0.045	0.964
Income level					
<2000	—	—	—	—	—
2000~5000	0.261	0.540	0.028	0.483	0.629
5001~10,000	0.125	0.707	0.009	0.177	0.859
>10,000	−0.242	1.289	−0.007	−0.188	0.851
Marital satisfaction	1.129	0.506	0.088	2.231	0.026
Past history of mood disorders	−0.602	1.075	−0.021	−0.560	0.576
First pregnancy	0.122	0.553	0.012	0.222	0.825
Previous miscarriage	−0.539	0.453	−0.058	−1.191	0.234
Parity	−0.123	0.453	−0.013	−0.271	0.787
Intimate partner violence	0.398	0.385	0.030	1.035	0.301
Obstetric complications	0.012	0.439	0.002	0.032	0.740
GAD-7	0.438	0.065	0.268	6.753	0.000
Positive coping	−0.560	0.245	−0.071	−2.282	0.023
Negative coping	0.491	0.229	0.063	2.147	0.032

GAD-7: Generalized Anxiety Disorder.
